# Supplementary material for: Multi-center prospective population pharmacokinetic study and the performance of web-based individual dose optimization application of intravenous vancomycin for adults in Hong Kong: A study protocol
Source: PLoS One. 2022 May 5;17(5):e0267894. doi: 10.1371/journal.pone.0267894 (PMC9070875; doi:10.1371/journal.pone.0267894)
Supplement: S1 File — (DOCX) [file pone.0267894.s001.docx]

Study Title: Multi-center population pharmacokinetic study of intravenous vancomycin in adults in Hong Kong and development of web-based individual dose optimization interface

Principle investigator: Dr. LAM Tai Ning, Teddy

Co-Investigators:

Prof. CHEUNG Yin Ting^1^, Dr. HUI Ka Ho, Matthew^1^, Dr. LUI Chung Yan, Grace^2,3^, Dr. LEE Kin Ping, May^2,4^, Dr. LI Chun Man Timothy^2,3^, Prof. WONG Wai Tat^2,5^, Prof. LING Ka Kin, Samuel^2,6^, Ms. TSANG Chui Shan, Rikki^2,7^, Ms. WONG Ting Yuk^2,7^

^1^School of Pharmacy, Faculty of Medicine, The Chinese University of Hong Kong (CUHK).

^2^Prince of Wales Hospital (PWH), New Territories East Cluster (NTEC), HA.

^3^Department of Medicine and Therapeutics, Faculty of Medicine, CUHK.

^4^Department of Microbiology, Faculty of Medicine, CUHK.

^5^Department of Anaesthesia and Intensive Care, Faculty of Medicine, CUHK.

^6^Department of Orthopaedics and Traumatology, Faculty of Medicine, CUHK.

^7^Pharmacy department, PWH, NTEC, HA.

1. Study Objective(s) and Significance

- Objective #1: To develop and validate a model characterizing the population pharmacokinetics (PK) (popPK) of intravenous (IV) vancomycin in Hong Kong adults using prospectively collected rich therapeutic drug monitoring (TDM) data from multiple hospitals under Hospital Authority (HA)
- Objective #2: To develop a web-based interface to enable Bayesian estimates of individual vancomycin exposure and evidence-based optimization of individual empirical and TDM-based dosing regimen
- Objective #3: To evaluate the clinical utility of the developed interface

The most recent treatment guideline recommends the use of TDM-based Bayesian estimation to promote the achievement of a balance between efficacy and toxicity of vancomycin in the treatment of *methicillin-resistant staphylococcus aureus* (MRSA) infection. PopPK modeling has been the cornerstone of obtaining accurate individual Bayesian estimates but has not been done in Hong Kong yet. Besides, Bayesian estimation involves numerical approximation procedures that cannot be simplified to simple equations nor nomograms for routine clinical reference. Thus, a validated computer program to perform Bayesian estimation and dose optimization is warranted to put this recommendation into practice. The development of a highly accessible web-based interface would be the best approach to maximize user-friendliness in the clinical setting. Successful achievements in this pilot study would greatly improve the confidence in IV vancomycin treatment, and perhaps more significantly, laid the foundation for the application of Bayesian dose individualization for many other pharmacological agents with narrow therapeutic indices.

1. Project Duration

May 1, 2021 to April 30, 2022 (12 months)

1. Background

Traditional TDM of IV vancomycin

In 2009, the American Society of Health-System Pharmacists (ASHP) guideline (the 2009 guideline) recommended a steady-state area under curve (*AUC*) over 24 hours (*AUC_24_*) to minimum inhibitory concentration (measured by broth microdilution, *MIC_BMD_*) ratio (*AUC_24_/MIC_BMD_*) of at least 400 as the PK target for adequate vancomycin efficacy. Nevertheless, in view of the difficulty to obtain multiple vancomycin concentrations (*C_s_*) for the estimation of *AUC*, ASHP recommended the use of steady-state trough *C_s_* (*C_s,ssTrough_*) as a surrogate marker for the *AUC_24_/MIC_BMD_* target.^1^

Updated guideline to TDM strategy

Over the past decade, the following changes and evaluations have led to the expert panel’s decision to update the guideline on TDM of vancomycin:

- Despite the previous recommendation, there has been minimal to no data on the efficacy and safety profile supporting the use of *C_s,ssTrough_* as a treatment endpoint.^2^
- There has been increasing evidence on the use of *AUC* as the therapeutic index of IV vancomycin.^3,4^
- Multiple studies done in the past decade led to the recommendation that when *MIC_BMD_* is measured to be 1 mg/L, which is usually the case, *AUC_24_* should be maintained between 400 and 600 mg·h/L to balance between efficacy and the risk of nephrotoxicity (mainly *acute kidney injury*, AKI).^2^
- Of equal importance, it has been reported that Bayesian software programs are able to accurately estimate *AUC_24_* based on trough *C_s_* (*C_s,Trough_*), although accuracy is even higher with both *C_s,Trough_* and peak *C_s_* (*C_s,Peak_*).^5^
- Rapid advancement in computing technology has greatly improved the access to Bayesian programs.

Combining the above evidence, ASHP updated the guideline in 2020 (the 2020 guideline) and now recommends:

- Bayesian estimation of individual *AUC* based on *C_s,Trough_* (or preferably, whenever feasible, both *C_s,Trough_* and *C_s,Peak_*, for better accuracy),
- promotion of the achievement of *AUC_24_/MIC_BMD_* therapeutic target of 400‑600 through dose individualization, and
- ***against*** the use of *C_s,ssTrough_* as a surrogate target.

Advantages of Bayesian estimation and popPK model

Since the therapeutic targets are based on observations at steady-state, it is often necessary to wait until the 3^rd^ or 4^th^ infusion (for intermittent infusion of vancomycin, II_vanco_), after which steady-state is usually reached, for the sampling of *C_s_*. But with Bayesian estimation, it is reliable to extrapolate the pre-steady-state PK profile to predict steady-state behavior. It implies that waiting until the steady state for sampling is no longer necessary, and *C_s_* sampled during the first 24 to 48 hours can be sufficient to inform the optimal steady-state dosing regimen.^2^ Moreover, thanks to its ability to extrapolate, Bayesian estimation can also be used to recommend the loading dose (in addition to the optimal maintenance dose) to further shorten the time required to reach desired exposure. The same set of individual Bayesian estimates can even be used to recommend the infusion rate in patients in dangerous situations where continuous infusion (CI_vanco_) is preferred over II_vanco_.

As mentioned in Section 1, a developed popPK model is required as the prior distribution for the execution of Bayesian estimation. The popPK model contains estimates of the effects of multiple covariates, including but not limited to age, sex, body weight (*WT*), and serum creatinine level (*S_Cr_*), on the PK of vancomycin. Therefore, Bayesian estimation is able to integrate the effects of multiple covariates, and on top of that, dynamically adapt to the changes in pathophysiological conditions of the patients during treatment when making updated dose recommendations. Besides, a popPK model also contains estimates of the variability of PK parameters within the study population. Bayesian estimation also takes this information into accounts and therefore is more likely to generate more accurate individual PK estimates by making use of and balancing the information gained by both population information and individual observed *C_s_*, in comparison to traditional PK estimation, which is only capable of taking individual observations into account.^6^

Current practice in Hospital Authority and recommendations

The TDM practice across institutions of HA to monitor *C_s_* has generally been in line with the 2009 guideline for the treatment of severe MRSA infection using IV vancomycin in that *C_s,ssTrough_* is being targeted. Nevertheless, TDM-based dosing adjustments have been empirical, without the collective information gained by individual patient’s *C_s_* measurement(s), age, sex, *WT*, and *S_Cr_*, and potentially other covariates of vancomycin PK, and without the help of Bayesian estimation. In view of the updated evidence on the therapeutic target of IV vancomycin and improved access to computing resources, we recommend advancing current practice to meet the suggestions by the 2020 guideline to enhance the achievements of desired treatment efficacy and toxicity profile.

Therefore, we set out the study objectives as stated in Section 1 above accordingly. It is expected that the study will bring about the following improvements in the effectiveness of IV vancomycin treatment:

- Eliminating the need for clinicians to guess the optimal dose of IV vancomycin by providing evidence-based dose recommendations
- Allowing empirical dose suggestion based on the popPK model developed
- Shortening the time to the achievement of the desired PK exposure by
- allowing the optimization of maintenance dose as soon as the first *C_s_* measured during the first 24 to 48 hours becomes available, and
- on top of the maintenance dose, suggesting the loading dose to promote rapid achievement of target, or when necessary, the infusion rate for CI_vanco_
- Taking into account the collective effects of multiple covariates on vancomycin PK
- Adapting to changes in patients’ pathophysiological conditions and updating the optimal dose accordingly to maintain the desired vancomycin exposure
- Given the above advantages, improving treatment outcome by balancing between efficacy and toxicity
- Enhanced clinician-friendliness by adopting a web-based graphical user interface
- Saving subscription fees to proprietary software by building the interface with open-source programs

1. Research plan and methodology

4a. Overview

Study design and population

This is a multi-center prospective study involving hospitals across all seven clusters of HA. All in-patient subjects (1) at least 18 years of age, (2) admitted to one of the following HA hospitals:

- HKEC: Pamela Youde Nethersole Eastern Hospital (PYNEH)
- HKEC: Ruttonjee and Tang Shiu Kin Hospitals (RTSKH)
- HKWC: Queen Mary Hospital (QMH)
- KCC: Queen Elizabeth Hospital (QEH)
- KCC: Kwong Wah Hospital (KWH)
- KEC: United Christian Hospital (UCH)
- KWC: Princess Margaret Hospital (PMH)
- NTEC: Prince of Wales Hospital (PWH)
- NTWC: Tuen Mun Hospital (TMH)

and (3) for whom intermittent IV vancomycin is prescribed, are eligible for recruitment into the study for data collection. The development of popPK model does not have definite requirements for minimum sample size but larger sample sizes are encouraged for more accurate parameter estimates. Based on the usual sample sizes of popPK studies and with respect to the expected availability of data from multiple hospitals, this study expects to recruit at least 300 subjects, which are likely feasible and adequate for robust model estimation. Among the dataset, 50 subjects will be set aside for external validation of the popPK model, while the rest belong to the model estimating set. At a later stage of the study, data from another 50 subjects will be collected for the evaluation of the performance of the developed interface.

Study procedure and expected timeline

It is expected that the popPK data collection period (PDCP) will last from May 2021 through Sep 2021. popPK model development will start around Sep 2021 once sufficient data have been collected for preliminary analyses. Background preparation work for the dose optimization interface will be in progress during the entire popPK study period. After the interface becomes ready-to-use, training to use the interface and evaluation of the performance of the developed interface are expected to start in Jan 2022.

4b. Research plan for objective #1

Data items to collect

For each subject, *constant* data items refer to variables that take only a single value. Meanwhile, all *dynamic* data items should be collected during each of his/her *individual data collection period(s)* (IDCP), defined as the period starting from *the start of the 1^st^ infusion* and until *the sampling for the last C_s_ measurement*. Data items required for the study are summarized as follows (see **Appendix 1** for details):

| Item type | | Data item |
| --- | --- | --- |
| Constant data items | | Date of birth |
|  |  | Ethnicity |
|  |  | Sex |
|  |  | Baseline *WT* |
|  |  | Body height (*HT*) |
|  |  | Obesity status |
|  |  | Baseline *S_Cr_* |
| Dynamic data items | Measurements, assessments, or events | Significant changes in *WT* (if any) |
|  |  | *S_Cr_* during treatment |
|  |  | Pathophysiological conditions: AKI, sepsis, *etc.* |
|  |  | Death (if applicable) |
|  |  | Concomitant drugs |
|  |  | Renal replacement |
|  | Vancomycin dosing records | Date and exact timing of start of infusion |
|  |  | Infusion rate |
|  |  | Total amount infused |
|  | *C_s_* | Date and exact timing of sampling |
|  |  | Measured concentrations |
|  | Microbial cultures | Date and time of sampling |
|  |  | Isolated micro-organisms |
|  |  | *MIC_BMD_* of vancomycin against MRSA |
|  | Time-to-event records | Time to achieving therapeutic target (*TTT*) |
|  |  | Time to recovery (*TTR*) |

Data collection method

Clinicians will order extra laboratory assays according to the data items required by the study protocol. Site investigators will collect and organize required data of recruited patient using the *Clinical Management System* (CMS), *Medication Administration Record* (MAR), *In-Patient Medication Order Entry System* (IPMOE), bedside patient chart, and, when necessary, verbal clarifications. *Anonymized* patient data will be shared in a confidential manner with the investigators in School of Pharmacy for data analyses. The hospital investigators shall keep a separate, confidential conversion list between anonymized identifiers (available to the investigators in School of Pharmacy) and on-site patient identifiers (e.g. HN no.), such that clarification will be possible when data validity is in doubt.

Data exclusion and management

Date in the following IDCP will be removed from subsequent analyses:

- IDCP with any missing constant data item (except *HT* unless subject is obese, and ethnicity),
- IDCP without baseline *S_Cr_*, or
- IDCP without at least *one* *C_s,peak_* (or random *C_s_*) plus at least *one C_s,trough_* sampled.

Besides, in each IDCP, all *C_s_* records that are over 168 hours (7 days) after the last *C_s_* record or the end of the last infusion (whichever later) will be removed from subsequent analyses.

Details of the imputation of *WT*, *S_Cr_*, and *HT* are available in **Appendix 2**. Preliminary analyses of individual *C_s_*-time profiles will be performed to identify potential outliers. When necessary, investigations into the possibilities of errors in data collection and unusual patient conditions causing the extreme values will be carried out as soon as possible.

PopPK model development and validation

- **Structural and parameter models**: The one- or two-compartment infusion model with first-order elimination will be tested, where the one demonstrating better goodness-of-fit (better *GOF*, demonstrated by smaller objective function value (*OFV*)) will be chosen. Between-subject and between-occasion variabilities in PK parameters are assumed to follow log-normal distributions. Residual unexplained variability will be described by a combined proportional-additive error model.^7-9^
- **Covariate model**: The effects of *WT* on PK parameters will be presumably estimated by the power model, where allometric scaling with pre-determined exponents will be tested against estimated exponents.^10^ Creatinine clearance will be approximated by the Cockcroft-Gault equation and associated with vancomycin clearance by testing different curve functions.^11^ Residual covariate effects are then tested against other potential covariates. Hypothesis testing at *α* = 0.01 will be conducted to compare the *GOFs* between two nested models by assuming that the change in *OFV* from the richer model to the sub-model follows the *χ^2^*-distribution with df = the number of constrained parameters.^12^
- **Model evaluation and validation**: Predictive plots, residual plots, normalized empirical Bayes estimates (*EBE*) plots, prediction-corrected visual predictive check (*pcVPC*), and normalized prediction distribution error (*NPDE*) will be inspected to evaluate the final model and parameter estimates.^13^ After the above evaluation, bootstrapping using 1,000 resamples will be done for internal validation. External validation will be done by evaluating the internally validated model against a separate, smaller dataset.
- **Software used**: NONMEM^®^ 7 will be used to obtain parameter estimates using a maximum likelihood estimation algorithm.^14^ Below-limit-of-quantification data will be assessed using the M3 method.^15^ Perl-speaks-NONMEM will be used to coordinate NONMEM^®^ runs and model evaluation.^16^ R and its packages will be used for the generation of model evaluation graphics.^17,18^

Study outcomes

In predictive plots, observations should scatter around the identity line. Weighted residuals and normalized *EBE* should scatter around zero (with 95% of the points lying within -1.96 and 1.96) with no observable trend alone and against time and other variables. pcVPCs should show general agreements between corrected observed and predicted *C_s_* in terms of the percentiles. *NPDE* plots should resemble the standard normal distribution. Bootstrapping should show reasonable distributions of bootstrap estimates with their medians close to and their 95% confidence intervals containing the model estimates. Apart from bootstrapping, the above applies also to external validation. Overall, all model diagnostics should indicate good predictive performance and stability of the developed PK model.

4c. Research plan for objective #2

Development of web-based individual dose optimization interface

The infrastructure and framework of a previously published web-based individual dose adjustment interface for high-dose methotrexate in the pediatric population will be replicated in this study. This interface was built using R and its packages, which are open sources, validated against the proprietary software package, NONMEM^®^, for the accuracy of individual parameter estimation, and shown to be more efficient than relying on NONMEM^®^ in performing individual parameter estimation.^19^ The interface will be amended to adapt to the requirements of this study and clinical application.

4d. Research plan for objective #3

Data items and collection

The data collection procedure at this stage of the study is very similar to that during the development of the popPK model, but may require sampling of *C_s,peak_* and *C_s,trough_* at a later stage of therapy to confirm target achievement at the steady state.

Study outcomes

- Primary outcomes focus on the achievement of the PK target of 400 ≤ *AUC_24_/MIC_BMD_* ≤ 600:
- The proportion of subjects achieving the PK therapeutic target at any time point during treatment (*P_target_*)
- *TTT*
- Secondary outcomes include
- the proportion of subjects developing *AKI* (*P_AKI_*)
- *TTR*

Statistical analyses

Several outcomes will be compared between two groups of subjects: (1) those recruited for popPK model development and (2) patients treated based on the usage of the developed interface. The *chi-square* *test* will be performed to test the null hypothesis that both groups have the same *P_target_*. The same applies to *P_AKI_*. *Kaplan-Meier analysis* and *Cox proportional hazard regression* will be used to describe *TTT* of the two groups and test the null hypothesis that both groups have the same *TTT*, respectively. The same applies to *TTR*.

4e. Ethical consideration

- **Need of patient data collection**: The current trough-based TDM of vancomycin is no more recommended due to its poor prediction of vancomycin exposure. A more reliable and currently recommended TDM strategy is to rely on Bayesian estimation of vancomycin AUC. The application of the strategy requires a prior distribution that is developed upon rich patient data. The results of the study enable HA institutions to comply with the most updated recommendations and is expected to improve treatment outcome once study results are applied.
- **Guideline-driven update in vancomycin TDM strategy**: Since the 2020 guideline from ASHP recommends against trough-only TDM strategy, this strategy should now be considered suboptimal. The bottom-line strategy, as recommended, should now consist of the measurements of at least one *C_s,trough_* and at least one *C_s,peak_*, regardless of the current study.
- **Extra procedure**: Based on the updated strategy, from the perspective of the patients recruited into this study, the study sampling schedule requires more measurements of *C_s_* than usual and thus will likely incur more frequent blood sampling than usual. From the perspective of the clinicians and helpers, workload may increase due to more frequent sampling, ordering of assays for extra samples, and collection and validation of detailed patient data.
- **Medical treatment**: There is no indication of medical treatment in this study. As in routine clinical practice, study subjects will receive medical treatments that are deemed the most appropriate by clinicians. It is well acknowledged that rich data collected in study subjects may alter clinicians’ decisions on vancomycin treatments. However, when compared to the current trough-only TDM strategy, the rich sampling scheme will likely enrich the information required for making accurate prediction of vancomycin exposure. Therefore, it is very unlikely that the extra procedures required in this study will adversely affect the optimality of therapy.
- **Patient privacy**: Access to identifiable patient data collected in this study will be available only to site investigators in each institution. All data will be anonymized before being sent to the data analyst. Data collected by the data analyst will be handled with encryption and password protection.
- **Informed consent**: The gathering of routinely collected data is not expected to adversely affect patient treatment nor expose patient information. However, since the rich sampling will incur extra procedures done on patients, an informed consent must be obtained from the patient or, if the patient is incapable of giving informed consent, his legal representative, who must be a member of his next of kin. For subjects who refuse to have extra procedures done on them, these extra procedures will not be performed and the data available through routine vancomycin treatment based on the updated guideline will continue to be included in this study.
- **Compliance to ethical standard**: This study will be conducted in compliance with the Declaration of Helsinki and is being submitted for review by Cluster Research Ethics Committees.

5. References

1. Rybak, M. et al. Therapeutic monitoring of vancomycin in adult patients: a consensus review of the American Society of Health-System Pharmacists, the Infectious Diseases Society of America, and the Society of Infectious Diseases Pharmacists. *American Journal of Health-System Pharmacy.* 66, 82-98 (2009).

2. Rybak, M. J. et al. Therapeutic monitoring of vancomycin for serious methicillin-resistant Staphylococcus aureus infections: A revised consensus guideline and review by the American Society of Health-System Pharmacists, the Infectious Diseases Society of America, the Pediatric Infectious Diseases Society, and the Society of Infectious Diseases Pharmacists. *Am J Health Syst Pharm.* 77, 835-864 (2020).

3. Jung, Y. et al. Area under the concentration-time curve to minimum inhibitory concentration ratio as a predictor of vancomycin treatment outcome in methicillin-resistant Staphylococcus aureus bacteraemia. *International journal of antimicrobial agents.* 43, 179-183 (2014).

4. Neely, M. N. et al. Prospective Trial on the Use of Trough Concentration versus Area under the Curve To Determine Therapeutic Vancomycin Dosing. *Antimicrobial Agents and Chemotherapy.* 62, e02042-02017 (2018).

5. Neely, M. N. et al. Are vancomycin trough concentrations adequate for optimal dosing? *Antimicrobial Agents and Chemotherapy.* 58, 309-316 (2014).

6. Fuchs, A., Csajka, C., Thoma, Y., Buclin, T. and Widmer, N. Benchmarking Therapeutic Drug Monitoring Software: A Review of Available Computer Tools. *Clinical Pharmacokinetics.* 52, 9-22 (2013).

7. Anderson, B. J., Allegaert, K., Van den Anker, J. N., Cossey, V. and Holford, N. H. G. Vancomycin pharmacokinetics in preterm neonates and the prediction of adult clearance. *British Journal of Clinical Pharmacology.* 63, 75-84 (2007).

8. Karlsson, M. O. and Sheiner, L. B. The importance of modeling interoccasion variability in population pharmacokinetic analyses. *Journal of Pharmacokinetics and Biopharmaceutics.* 21, 735-750 (1993).

9. Keizer, R. J., Karlsson, M. O. and Hooker, A. C. Modeling and simulation workbench for NONMEM: tutorial on Pirana, PsN, and Xpose. *CPT: pharmacometrics & systems pharmacology.* 2, 1-9 (2013).

10. Holford, N. H. G. and Anderson, B. J. Allometric size: The scientific theory and extension to normal fat mass. *European Journal of Pharmaceutical Sciences.* 109S, S59-S64 (2017).

11. Cockcroft, D. W. and Gault, M. H. Prediction of Creatinine Clearance from Serum Creatinine. *Nephron.* 16, 31-41 (1976).

12. Lewis, F., Butler, A. and Gilbert, L. A unified approach to model selection using the likelihood ratio test. *Methods in Ecology and Evolution.* 2, 155-162 (2011).

13. Nguyen, T. H. et al. Model Evaluation of Continuous Data Pharmacometric Models: Metrics and Graphics. *CPT: Pharmacometrics & Systems Pharmacology.* 6, 87-109 (2017).

14. Boeckmann, A. J., Sheiner, L. B. and Beal, S. L. *NONMEM Users Guides* (Icon Development Solutions, Ellicott City, MD, The United States, 2009).

15. Ahn, J. E., Karlsson, M. O., Dunne, A. and Ludden, T. M. Likelihood based approaches to handling data below the quantification limit using NONMEM VI. *Journal of Pharmacokinetics and Pharmacodynamics.* 35, 401-421 (2008).

16. Lindbom, L., Pihlgren, P. and Jonsson, E. PsN-Toolkit--a collection of computer intensive statistical methods for non-linear mixed effect modeling using NONMEM. *Computer Methods and Programs in Biomedicine.* 79, 241-257 (2005).

17. R Core Team *R: A language and environment for statistical computing* (2020) <<https://www.R-project.org/>>.

18. Wickham, H. *ggplot2: Elegant Graphics for Data Analysis* (Springer-Verlag New York, New York, NY, The United States, 2016).

19. Hui, K. H., Chu, H. M., Fong, P. S., Cheng, W. T. F. and Lam, T. N. Population Pharmacokinetic Study and Individual Dose Adjustments of High-Dose Methotrexate in Chinese Pediatric Patients With Acute Lymphoblastic Leukemia or Osteosarcoma. *Journal of Clinical Pharmacology.* 59, 566-577 (2019).

Appendix 1 – Data collection scheme

Data items to collect

For each recruited subject, the following *constant* data items (one value for each item for each subject) should be collected

- date of birth (or, if unavailable, year of birth)
- ethnicity
- sex
- baseline *WT* and *HT* (preferably (1) measured, or if infeasible, either (2a) a recent measurement reported by patient or based on institution record or (2b) a visual approximation, with an indication of the method used (1, 2a or 2b))
- (if *HT* is unavailable) whether subject is obese
- baseline *S_Cr_*

For each subject, all *dynamic* data items should be collected during each of his *individual data collection period(s)* (IDCP), defined as the period starting from *the start of the 1^st^ infusion* and until *the sampling for the last C_s_ measurement*. The most recent *C_s_* measurement can be considered the last one when (1) the subject has recovered and IV vancomycin treatment has been stopped, (2) the subject has been put off IV vancomycin and switched to receive alternative antibiotic(s), (3) the clinician has decided that IV vancomycin is to be halted indefinitely, or (4) the subject has deceased. (Note that either a temporary halt of IV vancomycin due to, for *e.g.*, impaired renal function, high *C_s_* *etc.*, or a change in IV vancomycin dosing regimen should *not* be considered an interruption of the IDCP.) If, within the PDCP, IV vancomycin is started on a subject *again* after his last IDCP, a new IDCP should be initiated for the same subject. If the PDCP ends before an IDCP of a subject, data collection for the subject should be extended until the IDCP ends as defined above. Dynamic data items required for the study are categorized and listed with details as follows:

- **(PopPK) dynamic data items with date and time of each of these measurements, assessments, or events**
- Significant changes in *WT* (if any)
- *S_Cr_* during treatment (preferably together with samples for *C_s_*, see below)
- Development and resolution of pathophysiological conditions:

*AKI*, sepsis, severe trauma, severe burns

- Death (if applicable)
- Starting and ending of concomitant drug treatments:

Diuretics, aminoglycosides, and non-steroidal anti-inflammatory drugs (NSAID)

- Starting and ending of renal replacement therapies: Dialysis and CRRT
- **(PopPK) dosing records**

For each infusion:

- The date and **exact** timing (*accurate to the minute* as much as possible) at the start of infusion
- The infusion period assuming constant-rate infusion (any irregularity in infusion rate and interruption must be documented with the timings of changes and rate changes)
- The total amount infused

In the case that it comes to the clinician’s or helper’s attention that the recruited subject either (1) has received IV vancomycin within 72 hours before the start of the 1^st^ infusion or (2) has severe baseline renal impairment and has received IV vancomycin within a week before the start of the 1^st^ infusion, then

- all available dosing records within these stated periods prior to the 1^st^ infusion should be recorded, and
- it should be noted whether such prior dosing records are complete or not.
- **(PopPK) vancomycin concentrations**

*C_s_* measurements with date and **exact** time (*accurate to the minute* as much as possible) of sampling documented. Subject to the actual implementation in individual hospital level, the recommended schedule of rich *C_s_* measurements required is as follows:

- *Two* peak levels (*C_s,peak_*): ***at least*** *one* hour and ***within*** *two* hours during dosing interval 1 (*C_s,peak1_*) and dosing interval 2 (*C_s,peak2_*)
- *Two* random levels (*C_s,random_*): ***at least*** an hour after the last *C_s,peak_* and ***at least*** an hour before the next *C_s,trough_* during dosing interval 1 (*C_s,random1_*) and dosing interval 2 (*C_s,random2_*)
- *Two* trough level (*C_s,trough_*): **within** *one* hour and ***strictly*** before the start of the *next* infusion during dosing interval 1 (*C_s,trough1_*) and dosing interval 2 (*C_s,trough2_*)

where dosing interval 1 refers to one of the pre-steady-state dosing intervals and dosing interval 2 refers to one of the steady-state or near-steady-state dosing intervals. In case it is infeasible to sample during a planned dosing interval, sampling should be delayed to the next feasible dosing interval. If vancomycin is put off before the last dose intended within the IDCP, then if vancomycin is restarted later, the schedule should be restarted from dosing interval 1. Note that any change in vancomycin dose and/or any supplementary vancomycin dose administered at once should not interrupt the planned sampling schedule. If there is a change in the administration frequency, the sampling schedule should be updated based on the new frequency. The timings of all samplings of *C_s_* after satisfying the above rich measurement schedule are not bound by the study protocol but subject entirely to clinical needs as judged by the clinicians.

In the case that not all the scheduled rich *C_s_* measurements can be performed (*e.g.* due to failure to obtain informed consent or change of treatment plan *etc.*), an alternative sparser schedule should be considered in the following order:

- *five*-sample schedule: *C_s,peak1_*+ *C_s,peak2_* + *C_s,trough1_* + *C_s,trough2_*+ (*C_s,random1_* or *C_s,random2_*)
- *four*-sample schedule: *C_s,peak1_*+ *C_s,peak2_* + *C_s,trough1_* + *C_s,trough2_*
- *three-*sample schedule: *C_s,peak1_*+ *C_s,peak2_* + *C_s,trough2_* or *C_s,peak2_*+ *C_s,trough1_* + *C_s,trough2_*
- *two-*sample schedule (same dosing interval): *C_s,peak1_*+ *C_s,trough1_* or *C_s,peak2_* + *C_s,trough2_*
- **(Outcome) microbial cultures**

For each sample sent for culture, the date and time of sampling, isolated microorganisms, and *MIC_BMD_* of vancomycin against MRSA.

- **(Outcome) Time-to-event records**

For each recruited subject:

- *TTT*
- *TTR*
- These records will also be collected from patients during the stage of evaluation of the performance of the developed interface to enable comparison with patients treated without the use of the interface.

Appendix 2 – Imputation of *WT*, *S_Cr_*, and *HT*

For each IDCP, *unique datetimes* (*t_unique_*) refer to the unique date-time combinations across all popPK dynamic data records, sorted in chronological order:

$\text{t}_{\text{unique}}=\left\{ \text{t}_{\text{1}} \text{, }\text{t}_{\text{2}} \text{,…,}\text{ }\text{t}_{\text{m}} \right\}$, where $\left\{ \begin{matrix} \text{m}\text{ }\text{=}\text{ }\text{total number of unique date-time combinations} \\ \text{t}_{\text{1}}\text{ }\text{<}\text{ }\text{t}_{\text{2}}\text{ }\text{<}\text{ }\text{…}\text{ }\text{<}\text{ }\text{t}_{\text{m}} \end{matrix} \right.$

Since *WT* and *S_Cr_* will not be present at all *t_unique_*, linear *interpolation* against time will be performed to fill *t_unique_* without *WT* or *S_Cr_* measurements. If the IDCP ends after the last measurement of *WT*, all *t_unique_* after the last measurement of *WT* (if any) will be filled by values imputed using linear *extrapolation* against time based on all *WT* measurements available within that IDCP. The same applies to *S_Cr_*. In the case that only the baseline *WT* measurement is available within an IDCP, all *t_unique_* within the IDCP will take the same *WT* value.

After the above procedure of filling in *WT* and *S_Cr_*, for each IDCP, *t_unique_* will be inspected for the time gap between all pairs of consecutive datetimes:

$\text{t}_{\text{gap,k}}=\text{t}_{\text{k+1}}-\text{t}_{\text{k}}$, where $\text{k}\text{ }\text{=}\text{ }\text{1,}\text{ }\text{2,}\text{ }\text{…,}\text{ }\text{m-1}$

For each time gap of *t_gap,k_* hour(s), a total of ⌊*t_gap,k_*⌋ dummy datetimes equally dividing the gap by time will be inserted, where *WT* and *S_Cr_* will be filled by linear interpolation. This step is to correct for the effects of continuously changing *WT* and *S_Cr_*, which is theoretically more realistic than assuming constant *WT* and *S_Cr_* between each consecutive pair of datetimes.

The same *HT* value will be assumed across all *t_unique_* within each IDCP. If *HT* is not present for an IDCP, multiple imputation will be used to impute the missing *HT* based on sex, age, and baseline *WT*.
