# Supplementary material for: Multi-center prospective population pharmacokinetic study and the performance of web-based individual dose optimization application of intravenous vancomycin for adults in Hong Kong: A study protocol
Source: PLoS One. 2022 May 5;17(5):e0267894. doi: 10.1371/journal.pone.0267894 (PMC9070875; doi:10.1371/journal.pone.0267894)
Supplement: S1 Data — (PDF) [file pone.0267894.s003.pdf]

知情同意書

詳細研究資訊

研究計劃標題：《靜脈注射萬古黴素於香港成年人中的多中心群體動力學研究及個人劑量最佳化網頁介面的開發》
主研究員：香港中文大學藥劑學院專業顧問，林泰寧博士
聯絡地址：新界沙田香港中文大學羅桂祥綜合生物醫學大樓八樓
聯絡電話: 3943 6827

參與者資料

姓名： ________________________________

性別：男/女 出生日期：______年______月______日

(一) 由參與者填寫

本人________________________________根據參與者資訊手冊的內容已 同意 / 不同意 參與
（參與者姓名）

本研究。________________________________已經對我解釋了參與本研究的各事項。
（研究員姓名）

我已充分的討論，並理解本研究的性質、目的、程序、潛在風險及效益。我已獲得一份參與者資訊手冊的副本，並有機會提出有關本研究的問題，也得到了滿意的回答和信息。
我了解我的參與是自願的，並可以在任何時候無理由退出參與。此舉將不會對我的醫療造成影響。我批准研究引用我的醫療記錄所含的信息。我了解我的姓名或其他個人資料在任何出版中將會被維持保密。

________________________________ ________________________
[參與者 簽名] （簽署日期）

(二) 由合法代理人填寫 (在適用情況下)

我 ________________________ 代表參與者同意參加本研究。我已得到了關於本研究的性質、
（合法代理人）
目的、程序、潛在風險及效益的清楚解釋，也已完全理解。

________________________________ ________________________
[合法代理人 簽名] （簽署日期）

(三) 研究員的聲明

我保證簽署本同意書的參與者或參與者的合法代表已得到關於參與研究的性質、目的、程序、潛在風險及效益的清楚解釋，並已明確理解。

________________________________ ________________________
（研究員簽名） （簽署日期）
