# Supplementary material for: Multi-center prospective population pharmacokinetic study and the performance of web-based individual dose optimization application of intravenous vancomycin for adults in Hong Kong: A study protocol
Source: PLoS One. 2022 May 5;17(5):e0267894. doi: 10.1371/journal.pone.0267894 (PMC9070875; doi:10.1371/journal.pone.0267894)
Supplement: S2 Data — (PDF) [file pone.0267894.s004.pdf]

參與者資訊手冊

您已被邀請參加一項研究。在您參加本研究之前，將有人向您說明，您也會有機會提問。請仔細閱讀以下內容所提供的信息。如您同意參加本研究，請簽署知情同意書。您將得到一份本文件的副本，以供您日後參考。

研究資訊

研究計劃標題:《靜脈注射萬古黴素於香港成年人中的多中心群體動力學研究及個人劑量最佳化網頁介面的開發》

主研究員: 香港中文大學藥劑學院專業顧問，林泰寧博士
聯絡地址：新界沙田香港中文大學羅桂祥綜合生物醫學大樓八樓
聯絡電話: 3943 6827

研究目的

「靜脈注射萬古黴素」(下稱萬古黴素，是一種抗生素藥物)是用於治療懷疑或確診「耐甲氧西林金黃色葡萄球菌」感染症的第一線藥物。雖然萬古黴素療效顯著，但由於其使用伴隨着導致急性腎衰竭的風險，治療期間必須進行「治療藥物監控」，即監控萬古黴素在人體中的份量，並在需要時調整萬古黴素的劑量以控制此份量，以平衡其療效及腎毒性。過去十多年來，控制目標以萬古黴素在血清中的濃度(下稱血藥濃度)作為基準，但最新研究顯示此目標的臨床效果未如理想。因此，美國衛生系統藥劑師協會在 2020 年間發表了報告，建議以「血藥濃度-時間曲線下面積」(下稱血藥面積)作為新的控制目標，以在療效及腎毒性間達致更佳的平衡。惟使用此新目標有兩個必要條件：(1)收集較多的血藥濃度數據，以建立一個適用於本地情況的群體藥物動力學模型，及(2)無法利用人手而必須依靠電腦作出運算。有見及此，這項研究旨在：(1)為香港成年人建立萬古黴素的群體藥物動力學模型，(2)開發自動化的個人劑量最佳化網頁介面予醫護人員使用，以及(3)探討使用此介面是否比使用舊控制目標更能達致療效及腎毒性間的平衡。

您是我們的研究對象，因為 (1) 您已年滿 18 歲，(2)您正在下列醫院管理局轄下的醫院接受治療：東區尤德夫人那打素醫院、律敦治及鄧肇堅醫院、瑪麗醫院、伊利沙伯醫院、廣華醫院、基督教聯合醫院、瑪嘉烈醫院、威爾斯親王醫院、或屯門醫院，並且(3)您的醫生已為您處方間歇靜脈滴注萬古黴素。本研究將在 1 年之內，在上述醫院招募總共至少 350 位意願者參加此研究。

研究過程

如果您同意參加本研究，您的醫護人員或會在您接受萬古黴素治療期間，為您採集**次數**比臨床需要為多(平均多 3 至 5 次)的血液樣本，並交回所屬醫院的微生物學部門測量血藥濃度。本研究並不會直接干涉您所接受的治療方案，治療期間您仍然會繼續接受您的醫生認為最適合您的治療安排。您的參與將於<u>採集了按照研究協議中所訂明的足夠的血藥濃度樣本</u> **或** <u>按照臨床需要採集了最後的血藥濃度樣本</u>(以較後者為準)時完結。除此之外，您的參與亦會在您的醫生認為您的臨床情況使您不適宜再繼續參與本研究時完結。

除了上述血藥濃度外，醫院的研究人員會在醫院的病人記錄中收集您在接受萬古黴素治療期間的其他臨床資料，以供研究分析所需，當中包括您的：出生日期、種族、性別、體重、身高、血清肌酸酐濃度(腎功能指標)、萬古黴素給藥記錄、其他病理情況、其他給藥記錄、細菌培養及藥物敏感試驗結果、達到控制目標的時間、以及康復時間。醫院的研究人員所收集的資料將會以絕對保密的形式送返香港中文大學藥劑學院，交由相關研究人員進行模型建立及分析。

研究中您的責任

如果您同意參與本研究，您應該：

- 執行以上所描述的研究程序，和跟從研究人員所給的建議，並且
- 如果您在研究過程中，有感覺任何不適的話，盡可能馬上通知您的醫生或研究人員。

退出參與研究

您可以在任何時間收回您的同意，並隨時中止參與研究，此舉動不會對您或您的醫療有任何影響。如果您（因任何理由）想中止參與研究，您需要立即通知您的醫生或研究人員。另外，因為以下原因，您的醫生、主研究員和本研究的研究人員也可能會隨時停止您參與研究：

- 不按照研究人員所給的指示
- 主研究員決定繼續參與研究的話會有不利
- 本研究被取消
- 其他行政原因
- 未預料到的情況

可能的風險，不適和不便

由於本研究可能涉及更頻密地測量血藥濃度，您的醫生可能會對您的萬古黴素的治療方案（例如劑量）作出與此相應的調整。但由於更頻密的測量一般來說會有助更精確地估計最適用於你的治療方案，我們預期此舉不會因此而對您產生任何嚴重的傷害。較頻密的抽血程序可能會令你感到少許額外的不適或不便(包括暫時性的疼痛和青紫)。如果您對於因本研究而採取的額外的抽血程序感到不舒服，您可以告知您的醫生，選擇不參與該次抽血。

潛在效益

對您個人來說，通過更頻密地測量血藥濃度，您的醫生或者可以更準確地估計您的身體情況，亦能按此調整您的治療方案。此舉或許可以提昇萬古黴素對您的病情的療效及/或降低其導致急性腎衰竭的風險。此外，您的參與也可能協助我們提昇萬古黴素於本地成年人群體中的治療效益，讓我們更加完善地為其他病人提供服務。

您的權利

您參與這項研究完全是自願性的。如果您有疑問，我們將會給您清楚和滿意的回答。如果有任何新的信息，會對參與此研究的情願有相關的話，主研究員或者他的代表人員會通知您或者您的合法代表人。通過簽署知情同意書，您仍未放棄任何您的法律權利，並可隨時撤銷您的同意和退出參與。

研究和醫療記錄保密

這項研究收集的所有病人資料均會被保密。您所提供的資料，在相關的法律和法規的範圍內，將不會公開。只有本研究的研究人員能夠得到所收集的保密資料，而在本研究中擔任資料分析的研究人員則只能夠得到被匿名處理後的病人資料。然而，通過簽署知情同意書後，研究人員和香港中文大學–新界東醫院聯網臨床研究倫理聯席委員會有權在保密的情況下直接取得您的病歷和醫療記錄正本以用來檢查研究程序和資料。此外，在任何有關此研究的公開發表中，您

的個人身份將不會被公開。除上述情況以外，除非得到您的准許，否則在本研究中所收集的您的個人資料將不會向第三方洩露。所有個人資料將於本研究完成後被保存為期最長兩年。

相關傷害與補償

醫院並不會彌補因研究造成的傷害。如果因非疏忽導致的未預料的傷害，賠償將會以個別案例來評估。但是，通過簽署此同意書，您仍未放棄任何您的法律權利或豁免涉及研究的各方的疏忽。

聯系

如果您有任何有關本研究和在本研究中您的權利 或 研究過程中造成傷害的問題，您可以致電 3943 6827 聯繫主研究員林泰寧博士。

如果您有任何有關本研究或您作為參與者的權利的問題，請致電 3505 3935 聯繫香港中文大學–新界東醫院聯網臨床研究倫理聯席委員會。
