# Supplementary material for: Multi-center prospective population pharmacokinetic study and the performance of web-based individual dose optimization application of intravenous vancomycin for adults in Hong Kong: A study protocol
Source: PLoS One. 2022 May 5;17(5):e0267894. doi: 10.1371/journal.pone.0267894 (PMC9070875; doi:10.1371/journal.pone.0267894)
Supplement: S3 Data — (PDF) [file pone.0267894.s005.pdf]

Informed consent form

Study details

Study title: *Multi-center population pharmacokinetic study of intravenous vancomycin in adults in Hong Kong and development of web-based individual dose optimization interface*

Principal investigator: Dr. LAM Tai-Ning, Teddy (Professional Consultant, School of Pharmacy, The Chinese University of Hong Kong)
Address: 8th Floor, Lo Kwee-Seong Integrated Biomedical Sciences Building, The Chinese University of Hong Kong, Shatin, New Territories
Telephone: 3943 6827

Participant particulars

Name: ________________________________

Sex：Male/Female Date of birth：__________________________ (dd/mm/yyyy)

1. Filled in by the participant

I, _______________________________, agree / disagree to participate in the study based on the
(name of participant)
information in the information for participant. _______________________________ has explained
(name of investigator)
to me the matters of participating in this study.

I have sufficiently discussed and understand the nature, purpose, procedures, and potential risks and benefits of this study. I have obtained a copy of the information for participant, and have had the opportunity to raise question(s) related to this study and obtained satisfactory answers and information.
I understand that my participation is voluntary, and that I can terminate my participation at any time without any reason, which will not affect the medical services I receive in any way. I grant the right for the study to quote the information contained in my medical records. I understand that my name and other personal information will be kept confidential in any publication.

___________________________________ ________________________
[Signature of participant] (Date of signature)

2. Filled in by legal representative (if applicable)

I, _____________________________, represent the participant to agree to participate in this study.
(name of representative)
I have been clearly explained the nature, purpose, procedures, and potential risks and benefits of this study, and I fully understand them.

_____________________________________ _______________________
[Signature of legal representative] (Date of signature)

3. Investigator declaration

I guarantee that the participant or his/her legal representative signing this consent form has been clearly explained the nature, purpose, procedures, and potential risks and benefits of the study, and they fully understand them.

_______________________________ ______________________
[Signature of investigator] (Date of signature)
