# Supplementary material for: Multi-center prospective population pharmacokinetic study and the performance of web-based individual dose optimization application of intravenous vancomycin for adults in Hong Kong: A study protocol
Source: PLoS One. 2022 May 5;17(5):e0267894. doi: 10.1371/journal.pone.0267894 (PMC9070875; doi:10.1371/journal.pone.0267894)
Supplement: S4 Data — (PDF) [file pone.0267894.s006.pdf]

INFORMATION FOR PARTICIPANT

You are invited to participate in a research study. Before your participation, someone will explain to you and you may ask questions about the study. Please carefully read the following information. If you agree to participate in the study, please sign the informed consent form. You will be given a copy of this document for your reference later.

STUDY INFORMATION

Study title: *Multi-center population pharmacokinetic study of intravenous vancomycin in adults in Hong Kong and development of web-based individual dose optimization interface*
Principal investigator: Dr. LAM Tai-Ning, Teddy (Professional Consultant, School of Pharmacy, The Chinese University of Hong Kong)
Address: 8th Floor, Lo Kwee-Seong Integrated Biomedical Sciences Building, The Chinese University of Hong Kong, Shatin, New Territories
Telephone: 3943 6827

PURPOSE OF THE STUDY

Intravenous vancomycin ("vancomycin", an antibiotic) is a medicine used in the first-line treatment of suspected or confirmed infection by the strain of bacteria called *methicillin-resistant staphylococcus aureus*. Although the efficacy of vancomycin is promising, it is known that its use is associated with the risk of developing acute kidney failure. Therefore, it is mandatory to perform *therapeutic drug monitoring* during the treatment, meaning to monitor the amount of vancomycin present in the human body and control this amount by adjusting the dose, in order to balance efficacy against kidney toxicity. In the past decade, the target has been to control the serum concentration of vancomycin (Cs). However, recent studies show that the clinical outcome of this target is not satisfactory. As a result, the American Society of Health-System Pharmacists published a report in 2020, recommending the use of the area under the Cs-time curve (AUC) as the new target to control to improve the balance between efficacy and kidney toxicity. Nevertheless, using the new target has two criteria: (1) collection of more Cs data than usual to build a population pharmacokinetic model that well describes the local population, and (2) the use of computer to perform calculation because manual calculation is not possible. For the above reasons, this study aims to (1) build a population pharmacokinetic model of vancomycin for Hong Kong adults, (2) develop a web-based automated individual dose optimization interface for use by healthcare professionals, and (3) investigate whether using the developed interface can strike a better balance between efficacy and kidney toxicity when compared to the previous target.

You are considered eligible as a research subject of the study because (1) you are at least 18 years old, (2) you are currently being treated at the following hospitals under Hospital Authority: Pamela Youde Nethersole Eastern Hospital, Ruttonjee and Tang Shiu Kin Hospitals, Queen Mary Hospital, Queen Elizabeth Hospital, Kwong Wah Hospital, United Christian Hospital, Princess Margaret Hospital, Prince of Wales Hospital, or Tuen Mun Hospital, and that (3) your doctor has prescribed intermittent intravenous vancomycin infusion for you. This study will recruit at least 350 volunteers in the above hospitals in a year.

STUDY PROCEDURES

If you agree to participate in this study, your healthcare providers *may*, during your treatment with vancomycin, perform blood sampling that is *more frequent* than is required clinically (3-5 times more on average). The collected samples will be returned to the microbiology department of the hospital for measurements of Cs. This study will not directly intervene the treatment that you receive. You will continue to receive treatments that are deemed the most appropriate by your doctor. Your participation in the study will terminate *when sufficient (according to the study protocol) Cs samples have been collected* **OR** *when the last clinically indicated Cs sample has been collected*, whichever is later. Apart from that, your participation will also be terminated when your doctor determines that you are no longer clinically suitable to continue the participation.

Apart from the Cs sample as mentioned above, the investigators in the hospital will collect other clinical data required for study analyses during your treatment with vancomycin from patient records. These include your: date of birth, race, sex, body weight, body height, serum creatinine level (which is an indicator of kidney function), vancomycin administration records, other pathophysiological conditions, other medication administration records, bacterial culture and results of drug sensitivity analyses, time of achieving the target, and time of recovery. The investigators in the hospital will forward the collected data, in a strictly confidential manner, to responsible investigators in School of Pharmacy, The Chinese University of Hong Kong, to carry our model development and analyses.

YOUR RESPONSIBILITIES IN THE STUDY

If you agree to participate in this study, you should：

- perform the study procedures described above and follow the recommendations by the investigators, and
- notify your doctor or investigators as soon as possible if you feel any discomfort during the study.

QUITTING THE STUDY

You have the right to withdraw your consent at any time and terminate your participation in the study. This will not affect you and the medical services you receive in any way. If you, for whatever reason, would like to terminate your participation in the study, you need to tell your doctor or investigator immediately. Besides, your doctor, the principal investigator, and study investigators may also terminate your participation at any time for the following reasons:

- the instructions given by the investigators are not being followed,
- the principal investigator decides that continued participation is detrimental,
- this study is cancelled,
- other administrative reasons, or
- unforeseeable circumstances.

POSSIBLE RISKS, DISCOMFORT, AND INCONVENIENCE

Since this study involves more frequent measurements of Cs, your doctor may adjust your vancomycin treatment (e.g. dose) accordingly. But since more frequent measurements generally help better estimate the optimal treatment plan for you, we do not expect this to cause any significant harm to you. More frequent blood sampling procedures may cause you some extra discomfort or inconvenience (e.g. temporary pain and bruises). If you feel uncomfortable about the extra blood sampling procedures incurred by this study, you may, at your discretion, notify your doctor and choose not to participate in a particular blood sampling procedure.

POTENTIAL BENEFITS

From your perspective, by measuring Cs more frequently, your doctor may be able to estimate your body condition more accurately and adjust your treatment accordingly. This may enhance the efficacy of vancomycin for your treatment and/or reduce its risk of leading to acute kidney failure. Besides, your participation may help us improve the overall efficacy of vancomycin in the local adult population, and let us serve other patients more comprehensively.

YOUR RIGHTS

Your participation in this study is entirely voluntary. We will answer clearly and satisfactorily should you have any enquiry. In case there is any new information that is pertaining to the willingness of

participating in this study, the principal investigator or his representative will inform you or your legal representative. Note that by signing the informed consent form, you have not given up any of your legal rights and you still have the right to withdraw your consent and terminate your participation in this study at any time.

CONFIDENTIALITY OF STUDY AND MEDICAL RECORDS

All patient data collected in this study will be kept confidential. Unless as indicated by related laws and regulations, the information that you provide will not be made public. Only investigators of this study can access the confidential data obtained, while investigators responsible for data analyses of this study can only access anonymized patient data. Nevertheless, by signing the informed consent form, investigators and The Joint Chinese University of Hong Kong - New Territories East Cluster Clinical Research Ethics Committee have the rights to directly obtain, in a confidential manner, the original copies of your medical history and records for the purpose of investigating the study procedures and information. Besides, your identity will not be made public in any publication related to this study. Except in the aforementioned situations, all of your personal information collected in this study will not be disclosed to any third party unless with your permission. All personal data will be kept for a maximum of 2 years upon the completion of the study.

RELATED HARMS AND COMPENSATION

The hospital will not compensate for any study-related harm. For any unforeseen harm that is not due to negligence, compensation will be assessed on a case-by-case basis. Nevertheless, by signing the informed consent form, you have not given up any of your legal rights and exempted negligence by parties of the study.

CONTACTS

Should you have any enquiry regarding this study, your rights in this study, or harm done during the study procedure, you can contact the principal investigator, Dr. LAM Tai-Ning, Teddy at 3943 6827.

If you have any enquiry regarding this study or your rights as a participant, you can contact The Joint Chinese University of Hong Kong - New Territories East Cluster Clinical Research Ethics Committee at 3505 3935.
