# Supplementary material for: Multi-center prospective population pharmacokinetic study and the performance of web-based individual dose optimization application of intravenous vancomycin for adults in Hong Kong: A study protocol
Source: PLoS One. 2022 May 5;17(5):e0267894. doi: 10.1371/journal.pone.0267894 (PMC9070875; doi:10.1371/journal.pone.0267894)
Supplement: Attachment — Submitted filename: Responses_to_reviewers_v1.1.docx [file pone.0267894.s007.docx]

Dear Editor,

All authors and I would like to thank the editorial staff and reviewers for handling our manuscript. Please see the following point-to-point responses to the editors’ and reviewers’ comments. Editors’ and reviewers’ comments are quoted in smaller grey font with tight line spacing, while our responses are in blue.

Journal Requirements:

When submitting your revision, we need you to address these additional requirements.

1. Please ensure that your manuscript meets PLOS ONE's style requirements, including those for file naming. The PLOS ONE style templates can be found at

https://journals.plos.org/plosone/s/file?id=wjVg/PLOSOne_formatting_sample_main_body.pdf and

https://journals.plos.org/plosone/s/file?id=ba62/PLOSOne_formatting_sample_title_authors_affiliations.pdf

Thank you for the information. We have reformatted our manuscript accordingly.

2. In your Data Availability statement, you have not specified where the minimal data set underlying the results described in your manuscript can be found. PLOS defines a study's minimal data set as the underlying data used to reach the conclusions drawn in the manuscript and any additional data required to replicate the reported study findings in their entirety. All PLOS journals require that the minimal data set be made fully available. For more information about our data policy, please see http://journals.plos.org/plosone/s/data-availability.

Upon re-submitting your revised manuscript, please upload your study’s minimal underlying data set as either Supporting Information files or to a stable, public repository and include the relevant URLs, DOIs, or accession numbers within your revised cover letter. For a list of acceptable repositories, please see http://journals.plos.org/plosone/s/data-availability#loc-recommended-repositories. Any potentially identifying patient information must be fully anonymized.

Important: If there are ethical or legal restrictions to sharing your data publicly, please explain these restrictions in detail. Please see our guidelines for more information on what we consider unacceptable restrictions to publicly sharing data: http://journals.plos.org/plosone/s/data-availability#loc-unacceptable-data-access-restrictions. Note that it is not acceptable for the authors to be the sole named individuals responsible for ensuring data access.

We will update your Data Availability statement to reflect the information you provide in your cover letter.

The protocol does not report any result. We have specified so in the Data Availability section.

3. Your abstract cannot contain citations. Please only include citations in the body text of the manuscript, and ensure that they remain in ascending numerical order on first mention.

Our abstract does not contain any citation.

4. Please include captions for your Supporting Information files at the end of your manuscript, and update any in-text citations to match accordingly. Please see our Supporting Information guidelines for more information: http://journals.plos.org/plosone/s/supporting-information.

We have added the caption for our Supporting Information files at the end of our manuscript and updated any in-text citations accordingly.

Reviewer #1: This is an interesting PK study that primarily describes a carefully planned PK analysis. The statistician is clearly an expert in NONMEM7. Although I am a statistical reviewer, I am more concerned that the paper is primarily written about the statistical procedures rather than the design of the study itself.

Thank you for your time reviewing our manuscript and your valuable comments. We understand that our current manuscript is lacking certain details regarding study design. Please see our responses below.

Some of the omissions that I see are:

--multi-center studies should describe how each center's personnel will be trained on the protocol so that patient procedures are homogeneous

We will explain the protocol thoroughly to the site coordinators, answer all questions they have before study commencement, and closely monitor the recruitment of and study procedures done on the first subject at each center. The section “Compliance to ethical standard, registration, and approval” has been supplemented.

--A lot of data will be collected, and this is well-described, but its relation to the study objectives is less clearly described.

Thank you for your reminder. We have supplemented the “Structural and parameter models” and “Covariate model” sections to explain how the data help characterize the concentration-time profiles of IV vancomycin, considering potential covariate effects.

--There is no sample size justification. Why 300? Why 50?

Unlike hypothesis testing, where the minimum sample size required can be approximated based on the expected effect size and the power needed, in a popPK modeling study where the major covariates are also known a priori, there is no established way to calculate the minimum sample size. The sample sizes of previously published popPK models vary significantly, for e.g., 16 and 36 in *Hui K. H. et al (2019) DOI: 10.1002/jcph.1349*, 326 in *Francis J. et al (2019) DOI: 10.1128/AAC.01964-18*, and 1,151 subjects in *Stringer F. et al (2013) DOI: 10.1177/0091270012447121*. We notice that most popPK reports had sample sizes that range from 50 to 300. Therefore, we aim at the higher end of the range by defining a sample size of 300 and hope for more accurate estimates while avoiding further increasing the study burden.

Similarly, there is no established way to calculate the minimum sample size for external validation. Our decision to include 50 subjects for external validation was based on the observation that most popPK studies adopt a sample size for external validation that is about 15-30% of the sample size for model estimation. (300 * 17% = 50)

--Bayesian methods seem appropriate. Yet you are using t-tests and standard survival estimators for the comparison.

Bayesian methods are being applied to estimate the individual PK and thus optimal doses. However, successfully verifying the prior distribution to be used in the Bayesian estimation process does not imply that using the developed interface to guide dosing will improve the overall clinical outcome. Therefore, t-tests and Cox regression are proposed to compare the clinical outcomes of the two treatment approaches: with *vs* without the developed Bayesian application. These are statistical tests commonly used to test for treatment effects in conventional clinical studies.

--More on historical controls. How can we be certain that the historical controls are comparable?

We will verify the comparability through baseline characteristics comparison and, if necessary, propensity score matching. We have supplemented this under the sub-section “Statistical analyses for outcomes” in the Material and methods section.

--Far too many acronyms throughout the paper. I know PK people like acronyms, but it is overwhelming for the reader to keep track.

We apologize for using too many acronyms. We have removed most acronyms for technical terms (such as Cs,ss,trough) and those used sparingly in the updated manuscript.

--Details such as in lines 208-212 would be better shown in a figure or flow chart.

Thank you for your recommendation. We think it is a great idea to trim the passage to improve readability. We have added a figure to explain the ideal and alternative sampling schedules with respect to the dosing schedule.

--Remove the software used in the abstract. NONMEM is not a statistical procedure, it is a software designed to do certain modeling. It is better to say the statistical modeling technique rather than refer to the software.

We have removed NONMEM from the abstract and replaced it with non-linear mixed-effect modeling techniques.

--A sweeping conclusion that related all these NONMEM C_s analyses to clinical practice.

We have added a conclusive sentence at the end of the sub-section “PopPK model estimation and validation” in the Materials and methods section. Thank you for the recommendation.

Reviewer #2: The introduction needs to clarify the other recommendations and guidelines being used to guide the new study.

Thank you for your time reviewing our manuscript. We have further explained (1) the choice of a popPK model as the prior distribution for Bayesian estimation as a recommendation of the guideline.

Recommend addressing that reference method or “gold standard” for calculating MIC is called broth microdilution (BMD) and how that incorporates to the study.

Target range should be AUC:MIC ratio of 400-600.

We have included an explanation in the footnote of Table 1 about the methods used to measure MIC.

Reviewer #3: Multi-center prospective pharmacokinetic study and the performance of web-based individual dose optimization application of intravenous vancomycin for adults in Hong Kong: A study protocol

I commend the authors on this ambitious study to develop an algorithm for area under the curve based individual vancomycin dosing.

Thank you very much for your support of our work. Please see our responses to your valuable comments below.

Line 111: Please define “HA”

HA refers to Hospital Authority, the statutory body governing public hospitals in Hong Kong. The definition has been supplemented at its first appearance in the main text.

Line 137: explain methods for a priori sample sizes

Unlike hypothesis testing, where the minimum sample size required can be approximated based on the expected effect size and the power needed, in a popPK modeling study where the major covariates are also known a priori, there is no established way to calculate the minimum sample size. The sample sizes of previously published popPK models vary significantly, for e.g., 16 and 36 in *Hui K. H. et al (2019) DOI: 10.1002/jcph.1349*, 326 in *Francis J. et al (2019) DOI: 10.1128/AAC.01964-18*, and 1,151 subjects in *Stringer F. et al (2013) DOI: 10.1177/0091270012447121*. We notice that most popPK reports had sample sizes that range from 50 to 300. Therefore, we aim at the higher end of the range by defining a sample size of 300 and hope for more accurate estimates while avoiding further increasing the study burden.

Similarly, there is no established way to calculate the minimum sample size for external validation. Our decision to include 50 subjects for external validation was based on the observation that most popPK studies adopt a sample size for external validation that is about 15-30% of the sample size for model estimation. (300 * 17% = 50)

Table 1

Constant Data:

Consider staging acute kidney injury at baseline and throughout the treatment protocol (ie RIFLE, AKIN, or KDIGO). This can easily be calculated with proposed laboratory values to be collected and can further stratify patients adding greater depth and applicability to the web-based model.

Also, include source of infection

Dynamic Data Items:

Include renal based outcome (ie time to development of AKI, Time to ESRD, time to change in AKI stage, etc.)

We agree that adding AKI staging enables deeper analyses, such as the dose relationship of AKI. We have added a statement about conversion to AKI stages at the end of the sub-section “Data exclusion and management” and a description of corresponding statistical analyses in the sub-section “Statistical analyses for outcomes” under the Materials and methods section. Besides, we have added the item “source of infection” in Table 1. Similarly, we have included renal outcomes in terms of AKI staging in Table 1 and the sub-section “Statistical analyses for outcomes”.

Line 127: For patients with a new IDCP, please explain how this new IDCP will be treated during the data analysis. Will this be a unique dataset? Define the timeframe between cessation of vancomycin and a new IDCP.

The primary purpose of defining the IDCP is to ensure the necessary rich Cs sampling near the start of vancomycin treatment. The new IDCP will still belong to the same subject in the dataset. There will be no wash-out period between consecutive IDCPs – once a new IDCP is indicated to start due to vancomycin re-initiation, the remaining vancomycin estimated to be in the subject’s body from the doses in the previous IDCP will be carried over for the estimation of Cs in the subsequent IDCP(s). A sentence has been added to the end of the first paragraph of the sub-section ”Data exclusion and management” under the Materials and methods section.

Line 189: Define dosing interval.

Dosing interval has been defined later in the same paragraph. We apologize for putting the definitions after usage, thus causing confusion. We have moved the definition to the start of the paragraph.

Line 227: 168 hours is between 28 and 42 half-lives of vancomycin. This seems excessive. Consider reducing this time frame or explain why this time frame is chosen.

Thank you for pointing this out. The intention of introducing this time frame is to remove any Cs whose prediction might be inaccurate due to missing dosing records. (E.g. it is reasonable to suspect that there are missing dosing records when vancomycin remains detectable in a subject with normal renal function 1 week after the last dose.) It is true that 168 hours is typically 28 to 42 half-lives of vancomycin. However, this ratio is much reduced in subjects with profound renal impairment. Therefore, we would like to take a 7-day period as an approximated average. Besides, we will keep an eye on any significant underestimation for Cs recorded after a suspiciously long period after the last dose in case any missing dosing records are not being picked up by the above 7-day method.

Line 290: Define TTT, TTA, TTN

To reduce the use of acronyms, as suggested by other reviewers, we have removed the acronyms TTT (time to therapeutic target attainment), TTA (time to afebrile), and TTN (time to normal white blood cell count) from the text.
